# Natural Killer Cell Mediated Cytotoxic Responses in the Tasmanian Devil

**DOI:** 10.1371/journal.pone.0024475

**Published:** 2011-09-21

**Authors:** Gabriella K. Brown, Alexandre Kreiss, A. Bruce Lyons, Gregory M. Woods

**Affiliations:** 1 Menzies Research Institute Tasmania, University of Tasmania, Hobart, Tasmania, Australia; 2 School of Medicine, University of Tasmania, Hobart, Tasmania, Australia; University of Sheffield, United Kingdom

## Abstract

The Tasmanian devil (*Sarcophilus harrisii*), the world's largest marsupial carnivore, is under threat of extinction following the emergence of an infectious cancer. Devil facial tumour disease (DFTD) is spread between Tasmanian devils during biting. The disease is consistently fatal and devils succumb without developing a protective immune response. The aim of this study was to determine if Tasmanian devils were capable of forming cytotoxic antitumour responses and develop antibodies against DFTD cells and foreign tumour cells. The two Tasmanian devils immunised with irradiated DFTD cells did not form cytotoxic or humoral responses against DFTD cells, even after multiple immunisations. However, following immunisation with xenogenic K562 cells, devils did produce cytotoxic responses and antibodies against this foreign tumour cell line. The cytotoxicity appeared to occur through the activity of natural killer (NK) cells in an antibody dependent manner. Classical NK cell responses, such as innate killing of DFTD and foreign cancer cells, were not observed. Cells with an NK-like phenotype comprised approximately 4 percent of peripheral blood mononuclear cells. The results of this study suggest that Tasmanian devils have NK cells with functional cytotoxic pathways. Although devil NK cells do not directly recognise DFTD cancer cells, the development of antibody dependent cell-mediated cytotoxicity presents a potential pathway to induce cytotoxic responses against the disease. These findings have positive implications for future DFTD vaccine research.

## Introduction

The Tasmanian devil (*Sarcophilus harrisii*), the world's largest extant marsupial carnivore, is only found on the island of Tasmania, in Australia. An infectious cancer, known as devil facial tumour disease (DFTD), has recently emerged within the species. Spread of the disease has resulted in a severe population decline and may drive this unique species to extinction [1]. The principle mode of DFTD transmission is through biting [2], which is particularly common between Tasmanian devils during social interactions such as feeding and mating. The cancer establishes as an allograft [2] and the infected devil succumbs to the disease without evidence of an immune response to the tumour [3].

Genetics has shown that DFTD arose from a single original tumour [2], [4]. Since the initial immunohistochemical characterisation of DFTD cells by Loh and colleagues [5], DFTD was considered a cancer of neuroectodermal origin. Recent studies on the DFTD transcriptome have established that DFTD was derived from a Schwann cell in a founder animal [4].

As cancer is usually a disease that originates within one animal and only affects that animal, the emergence of a contagious cancer is extremely rare. For this to occur, a cancer must escape the host immune surveillance to avoid an antitumour response. This could arise in the absence of a competent antitumour immune response. Our previous studies indicate that Tasmanian devils have many competent immune responses [3], [6]. Despite this, Tasmanian devils are prone to developing a variety of neoplasms [7]. It is therefore possible that Tasmanian devils do not have competent antitumour responses. These are generally mediated by cytotoxic T lymphocytes (CTL) and natural killer (NK) cells. In this paper we present evidence that Tasmanian devils form competent NK cell-mediated cytotoxic immune responses against tumour cells.

## Materials and Methods

### Cell lines

Human K562 cells were originally sourced from the American Type Culture Collection (ATCC). The cells were maintained in RPMI culture medium (GIBCO, New York, USA) supplemented with 10% vol/vol heat inactivated foetal calf serum (Bovogen Biological, Victoria, Australia), 5 mM L-glutamine (Sigma Aldrich, Ayrshire, UK) and 100 IU of gentamicin sulfate (Pfizer, Western Australia, Australia) at 37°C in a humidified atmosphere of 5% CO_2_/95% air or cryogenically frozen at −80°C in RPMI culture medium containing 10% DMSO. Cells were pelleted for assay use by centrifuging at 240 *g* for 5 min. The identity of the cell line was verified using positive Glycophorin A labelling (data not shown), and also as target cells for human innate NK cell cytotoxicity (data not shown), a characteristic which is consistent with the original description of the K562 cell line by Lozzio and Lozzio, in 1979 [8].

DFTD cell lines were provided by A.-M. Pearse and K. Swift, Tasmanian Department of Primary Industries, Parks, Wildlife and Environment (DPIPWE). The cell lines were established from primary tumour biopsy samples taken under the approval of the Animal Ethics Committee of Tasmania's Park and Wildlife Services (permit numbers 33/2004–5 and 32/2005–6). The cells were maintained in RPMI culture medium at 35°C in a humidified atmosphere of 5% CO_2_/95% air. DFTD cells are strongly adherent and were dislodged by flushing with RPMI culture medium or with rubber scrapers. Cells were pelleted for assay use by centrifuging at 240 *g* for 7 min. Cell number and viability counts were performed using Trypan blue exclusion on an improved nuembauer Haemocytometer.

### Tasmanian devils

The experiments involving the use of Tasmanian devils were conducted under the approval of the University of Tasmania Animal Ethics Committee (permit number A0009215). The captive Tasmanian devils used in this study were fully adapted to captivity and housed in secure shelters under quarantine conditions in accordance with the ethics permit. The devils were fed a diet of native meat from disease free areas and their health was maintained by DPIPWE keepers and veterinarians.

Anaesthesia of the Tasmanian devil is required for blood collection and has been widely used by DPIPWEveterinarians.The vapour anaesthetic Isofluorane® is the agent of choice, given its short recovery period and fewer harmful side effects than other inhalation anaesthetics (reviewed in [9]). The Isofluorane gas was administered in oxygen at an approximate rate of 2 L/min via a mask. No adverse effects were recorded in the Tasmanian devils used in this study. All Tasmanian devils were anaesthetised and approximately 10 ml of blood was taken from the jugular vein. Up to 2 ml of blood from each sample was injected into clot activating tubes(Greiner Bio-one, Frickenhausen, Germany). The remainder was injected into lithium heparin anticoagulant tubes (BD Biosciences, New Jersey, USA). The samples were stored at room temperature until arrival at the laboratory (<24 hours). Samples were processed under sterile conditions.

### Immunisations and adjuvants

DFTD cells were harvested from culture then irradiated with 20 Gy of gamma radiation using a Varian Clinac 23-EX linear accelerator (Varian Medical Systems Inc., California, USA). The cells were pelleted, resuspended in PBS and combined with an equal volume of montanide adjuvant (Seppic, Puteaux, France). Two healthy female Tasmanian devils (Td 1 and Td 2) were injected with 10^8^ irradiated cells in a total volume of 1 ml, containing equal parts cell suspension and adjuvant, subcutaneously into the right shoulder, limiting the number of injection sites. A total of four doses was given at monthly intervals. Blood samples were collected 14 days after each injection.

K562 cells were harvested, resuspended in PBS and combined with an equal volume of montanide adjuvant. Four healthy female Tasmanian devils, (Td 3, Td 4, Td 5 and Td 6) were injected with 10^8^ cellsin a total volume of 1 ml, containing equal parts cell suspension and adjuvant, subcutaneously into the right shoulder. A total of two doses was given at monthly intervals. Blood samples were collected 14 days after each injection. Six months later, two devils (Td 3 and Td 6) were boosted with a third dose of K562 cells.

### Blood sample processing

Blood stored in clot-activating tubes was centrifuged at 1100 *g* for 10 minutes and the serum was harvested. The clot was removed and the process repeated.

Peripheral blood mononuclear cells (MNC) were isolated from uncoagulated whole blood using density gradient centrifugation on Histopaque 1077 solution according to the manufacturer's protocol (Sigma Aldrich, St Louis, USA). The MNC were washed with PBS at 250 *g*. The cells were diluted for assay use in culture medium.

### Separation of mononuclear cell populations using nylon wool adherence

As no methods were available for the specific isolation of cytotoxic cells in Tasmanian devils, T lymphocytes were enriched in MNC suspensions by depleting B lymphocytes using nylon wool adherence according to the previously published method [10]. Briefly, columns containing 0.6 g of nylon wool were saturated with RPMI culture medium and equilibrated at 37°C for 30 min and washed with RPMI culture medium. Mononuclear cell suspensions were applied to the columns.Small volumes of RPMI culture medium were added gradually, over a period of approximately 10 min. The eluent containing enriched T cells was centrifuged at 250 *g*. The cells were diluted for assay use in RPMI culture medium.

### Monocyte depletion using plastic adherence

Monocytes were depleted from mononuclear cell layers using plastic adherence, as described by Horowitz and colleagues [11]. MNC suspensions in RPMI culture medium were applied to the surface of 35 mm culture dishes (Iwaki, Tokyo, Japan), gently agitated to thinly cover the surface and incubated at 37°C for 45 min. RPMI culture medium was added dropwise and the dish gently agitated to loosen the plastic non adherent cells. The solution was collected and the wash repeated twice. The plastic non adherent cells were centrifuged at 250 *g*. The cells were diluted for assay use in culture medium.

### Leukocyte cytotoxicity assays

Cytotoxicity assays were performed using triplicate samples in V-bottomed 96 well plates (Greiner Bio-one, Frickenhausen, Germany). Effector ratios of 100∶1, 50∶1, 25∶1, 12∶1, 6∶1 and 3∶1 were tested against samples of 10^4^ target cells. Negative and positive cytotoxicity controls contained RPMI culture medium and 1% Triton X detergent in water, respectively. Cultured DFTD cells were incubated with 100 µCi of radioactive ^51^Cr solution (5 mCi/ml sodium chromate in normal saline – PerkinElmer, Massachusetts, USA) for 2 hr, with frequent gentle agitation. Cultured K562 cells were incubated with 100 µCi of radioactive ^51^Cr solution for 1 hr, with regular agitation. Labelled cells were washed 3 times in RPMI culture mediumthen diluted for assay use. The assays were incubated for 18 hr at 37°C in a humidified atmosphere of 5% CO_2_/95% air. The plates were centrifuged briefly at 170 *g* for 4 min then 100 µl aliquots of supernatant were harvested into polystyrene tubes and analysed for radioactivity (in counts per minute) using a Genesys gamma radiation counter (Laboratory Technologies Inc., Illinois, USA).

### Antibody-dependent cell cytotoxicity and Natural Killer cell assays

The procedure for lymphocyte cytotoxicity assays was modified to detect antibody-dependent killing. Triplicate samples of MNC, nylon wool non adherent cells or plastic non adherent cells at ratios of 25∶1, 12∶1, 6∶1 and 3∶1 were tested against samples of 10^4^ target cells.Serum from K562 immunised devils (Td 3 or Td 6 after a third injection) was diluted 1/10 in RPMI culture medium and 50 µl was added to the wells of test assays. Pre immune serum diluted 1/10 or RPMI culture medium was added to control assays. The assays were incubated for 18 hr before analysis. NK cell assays were performed with and without serum using the antibody-dependent cytotoxicity assay procedure but incubated for 4 hr before analysis.

### Formulae and statistics

Mean counts per minute (CPM) values were calculated from replicates and the percent cytotoxicity values were calculated according to the equation:
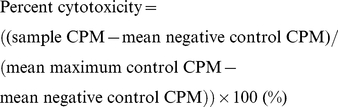
Statistical significance for chromium release data was calculated using an F test of pre-immune and post immune data sets for immunised devils or on serum free vs. serum supplemented samples for ADCC and 4 h NK assays. In assays involving nylon wool and plastic non adherent cells, F tests were performed between pre immune and non adherent cell data sets, then between total mononuclear cell and non adherent cell data sets to calculate statistical significance. Results were considered significant with a p value below 0.05 (*) and highly significant below 0.0005 (**).

### Measurement of serum antibody

Rabbit anti devil immunoglobulin (RαDIg) was purified using a protien A column (Sigma Aldrich, St Louis, USA) from the serum of rabbits immunised with Tasmanian devil whole serum. Tasmanian devil serum was diluted 1/25 in PBS. DFTD tumour cells were diluted to 5×10^6^ cells/ml and 100 µL aliquots were incubated with an equal volume of diluted serum for 20 minutes at 21°C. The samples were washed in PBS, with centrifugation at 14,000 *g* (in a microcentrifuge) for 1 minute. The samples were incubated and washed (as above) with RαDIg and then Alexa Fluor 488 goat anti rabbit IgG (Invitrogen, Oregon, USA). All samples were diluted to approximately 400 µL volume and analysed by flow cytometry on a BD Canto II (Becton Dickinson, New Jersey, USA) operating a 488 nm solid state laser. Although the parameters were adjusted for each sample, approximate voltages used on DFTD and K562 cells were 235 (forward scatter), 405 (side scatter) and 240 (Alexa 488).

### Immunocytochemistry and histology staining of MNC cytospins

MNC were diluted to 2×10^5^ cells/ml in PBS. Cytospins were prepared at 55 *g* for 5 min then immediately fixed in acetone. For giemsa staining, samples were covered in a modified giemsa solution designed for staining of cellular blood components and blood parasites (Fluka/Sigma Aldrich, St Louis, USA). The solution was filtered and diluted 1∶10 in phosphate buffered water (pH 6.5) prior to use. The samples were stained for 6 min then washed thoroughly.

For immunocytochemistry, peroxidase block (3% hydrogen peroxide in PBS) was applied to each cytospin for 15 min. This was followed by Dako's serum free protein block solution (Dako, California, USA) for 30 min. Rabbit anti-human CD3 (Dako, California, USA) and mouse anti-human MHC II (Dako, California, USA) primary antibodies were diluted in commercial diluent (Dako, California, USA) then applied to the cytospins for 4 hr at 21°C. Secondary anti-rabbit and mouse HRP linked secondary antibodies (Dako, California, USA) were applied to samples labelled with single antibodies and the LSAB universal link HRP system (Dako, California, USA) was applied to slides labelled with both antibodies. Finally, the samples were labelled with DAB chromogen (Dako, California, USA). The samples were counterstained in Mayer's hematoxylin (HD Scientific, New South Wales, Australia), mounted in aqueous medium (Dako, California, USA) and visualised under a light microscope (Olympus, Victoria, Australia) with mounted camera (Leica, Wetzlar, Germany).

## Results

### Tasmanian devils do not form cytotoxic responses or produce specific antibody after injection with irradiated DFTD cells

**Figure 1:**
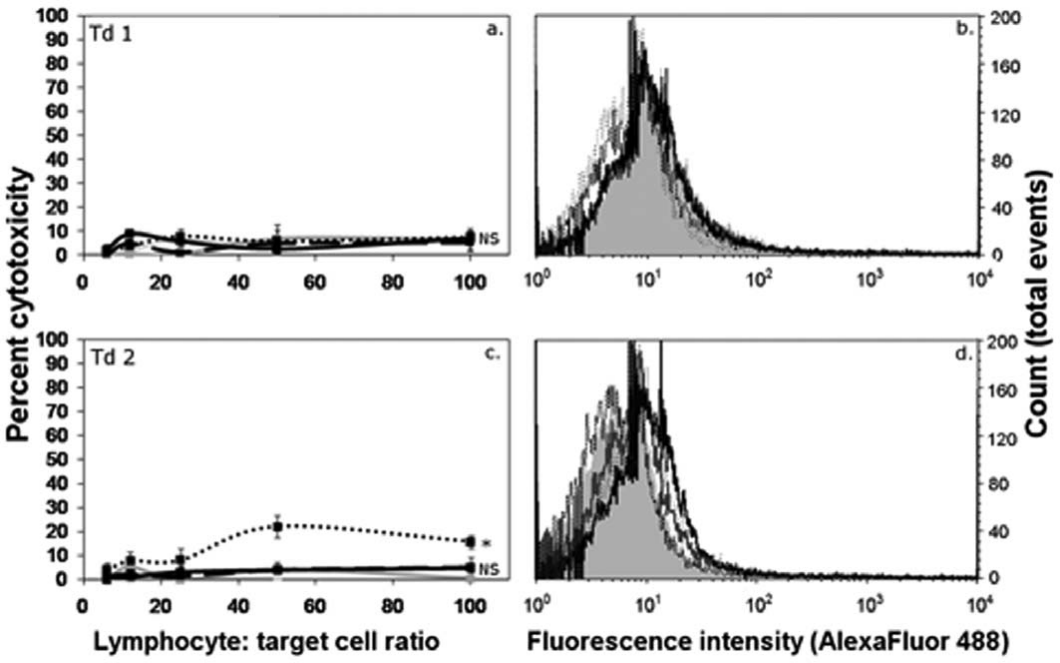
Cytotoxic and antibody responses against DFTD tumour cells. ^51^Cr release assays were performed for 18 hr at lymphocyte: target ratios of 100∶1, 50∶1. 25∶1. 12∶1, 6∶1 and 3∶1 (panels a and c). Pale grey lines represent pre-immune cytotoxicity levels. Responses after a single dose are shown as dark grey lines. Solid, dashed and dotted black lines represent the responses after two, three and four doses, respectively. One devil showed a weak increase in cytotoxicity after four doses, which was statistically significant compared to pre immune levels (F test; * p<0.05). Flow cytometry was performed to detect antibodies in devil serum (panels b and d). Neither devil produced antibodies against DFTD.

To induce anti-tumour responses against DFTD, two healthy captive Tasmanian devils were injected with irradiated DFTD cells and evidence for an immune response was evaluated by testing for anti-DFTD antibodies and cellular cytotoxicity. Prior to immunisations, there was no evidence for spontaneous or NK-like cytotoxicity against the DFTD cells, nor was there any evidence for the presence of anti-DFTD antibodies. After four immunisations, one devil produced a weak cytotoxic response against DFTD cells. However, there was no evidence of simultaneous antibody development (Fig. 1). The second devil did not produce cytotoxic responses or antibody after any dose.

### Tasmanian devils form cytotoxic responses and produce specific antibody after injection with xenogenic K562 tumour cells

**Figure 2:**
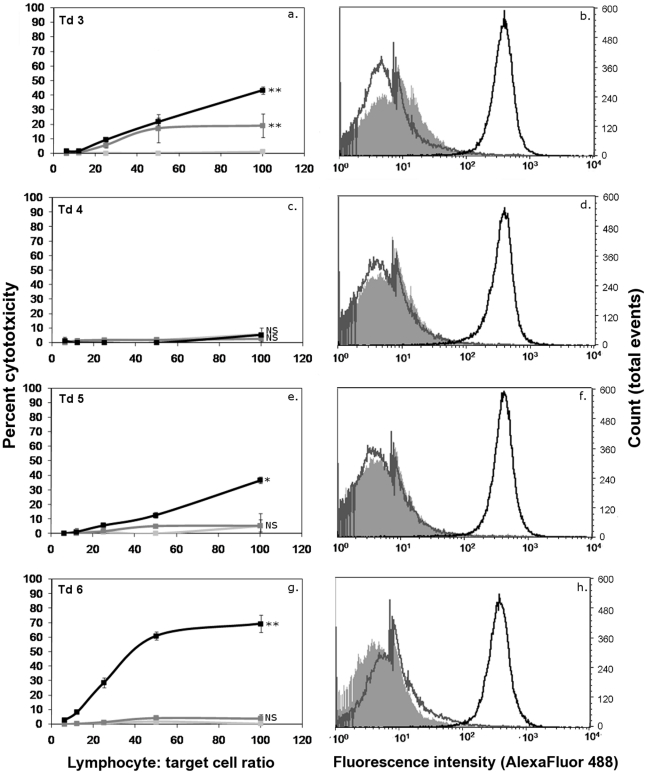
Cytotoxic and antibody responses against K562 tumour cells. ^51^Cr release assays were performed for 18 hr at lymphocyte: target ratios of 100∶1, 50∶1. 25∶1. 12∶1, 6∶1 and 3∶1 (panels a, c, e and g). Pale grey lines represent pre-immune cytotoxicity levels. Responses after a single dose are shown as dark grey lines. Black lines represent the responses after two doses. One devil developed a weak response after one dose (panel a). After two doses, three of the four Tasmanian devils formed cytotoxic responses against K562 cells. The levels were statistically significant compared to pre immune data (F test; * p<0.05, ** p<0.0005). Flow cytometry was performed to detect antibodies in devil serum (panels b, d, f and h). All devils formed strong antibody responses after two doses.

The inability of Tasmanian devils to produce cytotoxic responses and antibody against DFTD tumour cells may be due to a generalised incapacity to develop anti-tumour immunity. To examine the ability of Tasmanian devils to mount cytotoxic responses, four devils were injected subcutaneously with human K562 cells and evidence for an immune response was evaluated by testing for anti-K562 antibodies and cellular cytotoxicity. No spontaneous cytotoxicity was observed in the pre-immune samples, suggesting that no spontaneous NK-like killing was occurring. Three of the four devils formed cytotoxic responses after two doses of K562 cells (Fig. 2). All four devils formed strong antibody responses after two doses (Fig. 2). These data show that Tasmanian devils can mount functional antitumour responses against xenogenic cancer cells.

### Cytotoxic responses do not occur in nylon wool adherent cell depletedpreparations from K562 immunised Tasmanian devils

**Figure 3:**
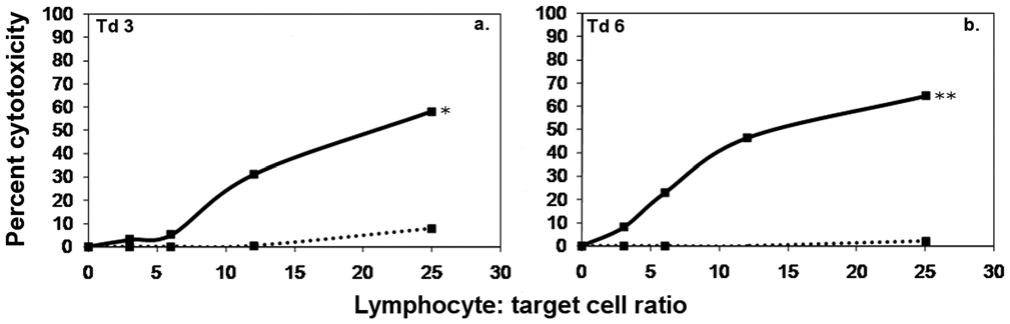
Cytotoxic responses of nylon wool non adherent cells from K562 immunised Tasmanian devils. ^51^Cr release assays were performed for 18 hr at lymphocyte∶ target ratios of 25∶1. 12∶1, 6∶1 and 3∶1. Solid black lines represent the responses of MNC. Dotted lines represent the responses of nylon wool non adherent cells. Cytotoxicity responses were absent in preparations of nylon wool filtered cells. The difference between nylon wool non adherent cells and mononuclear cell responses was statistically significant (F test; * p<0.05, ** p<0.0005).

Filtration of mononuclear cells through nylon wool is a widely used technique which can increase proportions of T lymphocytes in suspensions by removal of several other cell types, such as B lymphocytes and plasma cells [10]. When samples of filtered preparations were examined using CD3 immunocytochemistry, a high proportion of T lymphocytes was observed and labelling with MHC II showed that low numbers of B lymphocytes and few monocytes were present (Table 1). When the anti-K562 cytotoxic activity of nylon wool non adherent cells from two immunised devils was evaluated, no response occurred (Fig. 3). The total mononuclear cell layers of these samples formed strong responses.

**Table 1:** Percentages of leukocytes in Tasmanian devil peripheral blood mononuclear cell, nylon wool filtered and plastic non adherent cell populations.

Cell type	Cellular markers	Morphology	Percent total cell count		
			Mononuclear cells	Nylon wool filtered cells	Plastic non adherent cells
T lymphocyte	CD3^+^	Large nucleus, scanty cytoplasm, granules	55±8	73±7	76±12
B lymphocyte	MHC II ^+^	Large nucleus, scanty cytoplasm, no granules	33±8	9±6	13±4
Monocyte	MHC II ^+^	Bean shaped nucleus, abundant cytoplasm	5±3	4±2	1±1
NK-Like	CD3^−^/MHCII^−^	Large nucleus, scanty cytoplasm, granules	4±1	5±2	3±2
Neutrophil	CD3^−^/MHCII^−^	Large cell with multi lobed or ring shaped nucleus	7±5	13±10	4±3

### Naive Tasmanian devil lymphocytes form cytotoxic responses against K562 cells in the presence of anti K562 serum

**Figure 4:**
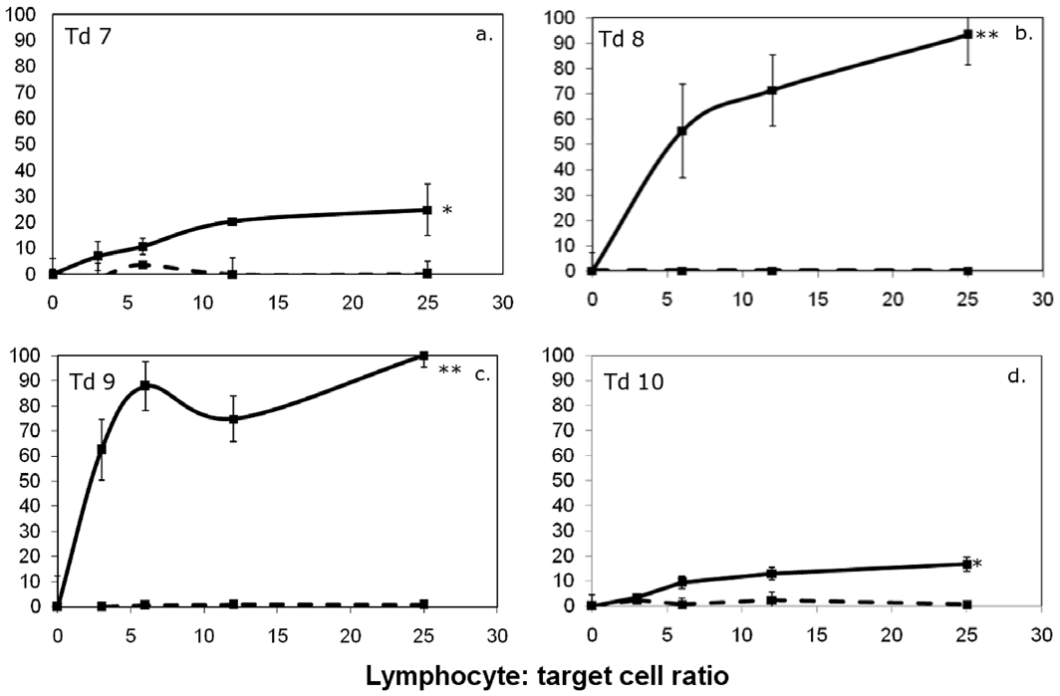
ADCC responses of MNC from naive Tasmanian devils against K562 cells. ^51^Cr release assays were performed for 18 hr at lymphocyte∶ target ratios of 25∶1, 12∶1, 6∶1 and 3∶1, in the presence or absence of immune serum. Solid black lines represent the responses of MNC in the presence of K562 immune serum. Dashed lines represent levels of cytotoxicity in naive MNC without serum. Addition of immune serum significantly increased cytotoxicity levels in all devils (F test; * p<0.05, ** p<0.0005).

Nylon wool filtrationwould be unlikely to remove cytotoxic effector cells [10], [12] butby depleting B lymphocytes and plasma cells it would remove the potential for antibody formation. If B lymphocytes and plasma cells produced antibody within the assays they may facilitate antibody dependent cell-mediated cytotoxicity (ADCC) against the tumour cells. MNC from naive devils formed cytotoxic responses against K562 cells in the presence of immune serum (Fig. 4).

### Nylon wool filtration does not deplete ADCC effector cells

**Figure 5:**
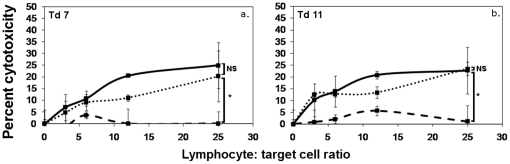
ADCC responses of nylon wool non adherent cells from naive devils against K562 cells. ^51^Cr release assays were performed for 18 hr at lymphocyte∶ target ratios of 25∶1, 12∶1, 6∶1 and 3∶1, in the presence or absence of K562 immune serum. Solid black lines represent the responses of MNC in the presence of immune serum. Dashed lines represent levels of cytotoxicity in naive MNC without serum. Dotted lines represent the responses of nylon wool non adherent cells in the presence of immune serum. In the presence of immune serum, there was no statistically significant difference between the responses of MNC and nylon wool non adherent cells. The responses of nylon wool non adherent cells in the presence of immunised serum were significantly greater than samples without immune serum (F test; * p<0.05).

ADCC responses can be mediated by cells such as monocytes and Natural Killer (NK) cells(reviewed in 13). Nylon wool filtration depleted numbers of monocytes and possibly other effector cells that may mediate the ADCC responses against K562 (Table 1). Serum from immunised devils was added to cytotoxicity assays performed with nylon wool non adherent cells from naive devils to determine if the effector cells remained after filtration. In the presence of immune serum,nylon wool non adherent cells formed cytotoxic responses (Fig. 5) indicating that theeffector cells are not removed by nylon wool filtration and are unlikely to be B lymphocytes or plasma cells.

### Plastic adherence does not deplete ADCC effector cells

**Figure 6:**
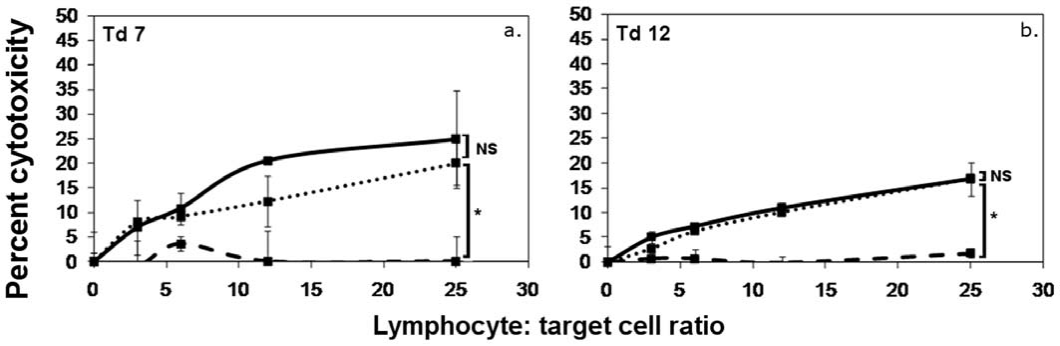
ADCC responses of plastic non adherent cells from naive devils against K562 cells. ^51^Cr release assays were performed for 18 hr at lymphocyte∶ target ratios of 25∶1, 12∶1, 6∶1 and 3∶1, in the presence or absence of K562 immune serum. Solid black lines represent the responses of MNC in the presence of immune serum. Dashed lines represent levels of cytotoxicity in naive MNC without serum. Dotted lines represent the responses of plastic non adherent cells in the presence of immune serum. There was no statistically significant difference between the responses of MNC and plastic non adherent cells in the presence of serum. The responses of plastic non adherent cells in the presence of immunised serum were significantly greater than samples without immune serum (F test; * p<0.05).

Monocytes are strongly adherent to plastic and were removed from MNC suspensions by plastic adherence [11]. The non adherent cells contained high proportions of T lymphocytes and only a few monocytes (Table 1). Plastic non adherent cells formed cytotoxic responses in the presence of immune serum (Fig. 6), indicating that monocytes are not the principal effectors of the ADCC responses.

### Presence of anti K562 serum antibody induced rapid NK-like cytotoxic responses

**Figure 7:**
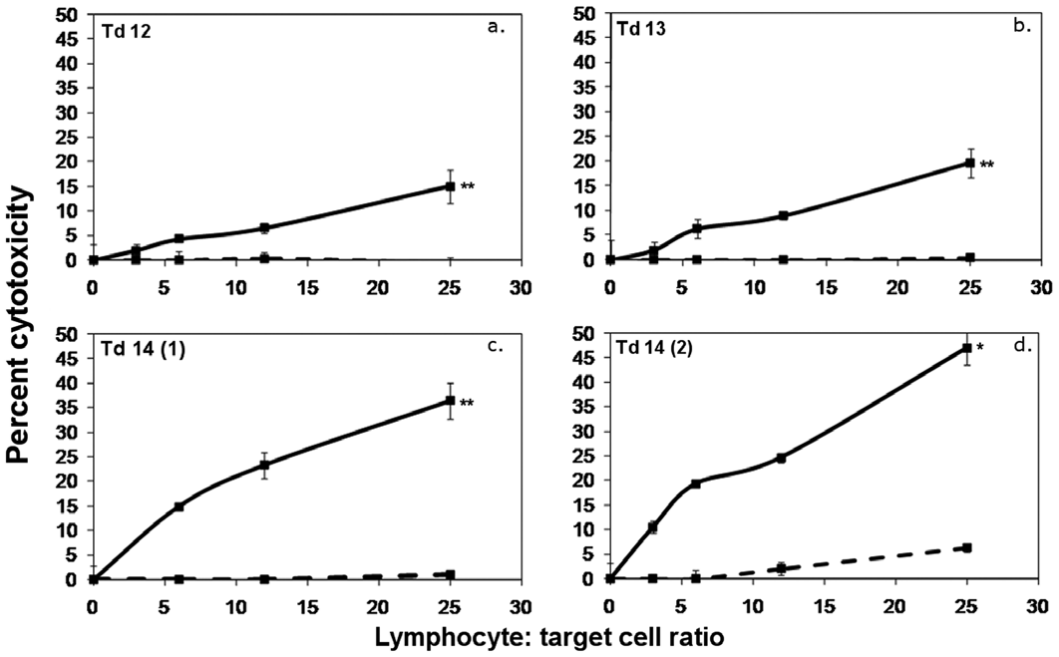
Natural Killer-like ADCC responses against K562 cells. ^51^Cr release assays were performed for 4 hr at lymphocyte∶ target ratios of 25∶1, 12∶1, 6∶1 and 3∶1, in the presence or absence of K562 immune serum. Solid black lines show the responses of naive MNC in the presence of immune serum. Dashed lines represent levels of cytotoxicity of naive MNC without serum. In the presence of immune serum and within four hours, MNC from naive Tasmanian devils consistently formed cytotoxic responses against K562 cells. One devil was tested twice and formed similar responses on both occasions (panels c and d). Addition of immune serum induced significant levels of cytotoxicity within four hours in all devils (F test; * p<0.05, ** p<0.0005).

As the ADCC responses against K562 cells occurred without the involvement of T lymphocytes, monocytes and neutrophils, it is possible that NK cells are the effectors. A distinguishing characteristic of NK cytotoxicity is a rapid response, and standard NK cell functional assays are performed over four-hour time periods [13]. Four hour cytotoxicity assays were performed with mononculear cells from naive devils and anti-K562 antibody. Cytotoxic responses were consistently formed within this time period (Fig. 7). One devil was tested twice, on different days, and produced strong responses in both assays. This is strong evidence for the functional presence of NK cells in Tasmanian devils.

### Cells with NK-like morphology are present in Tasmanian devils

**Figure 8:**
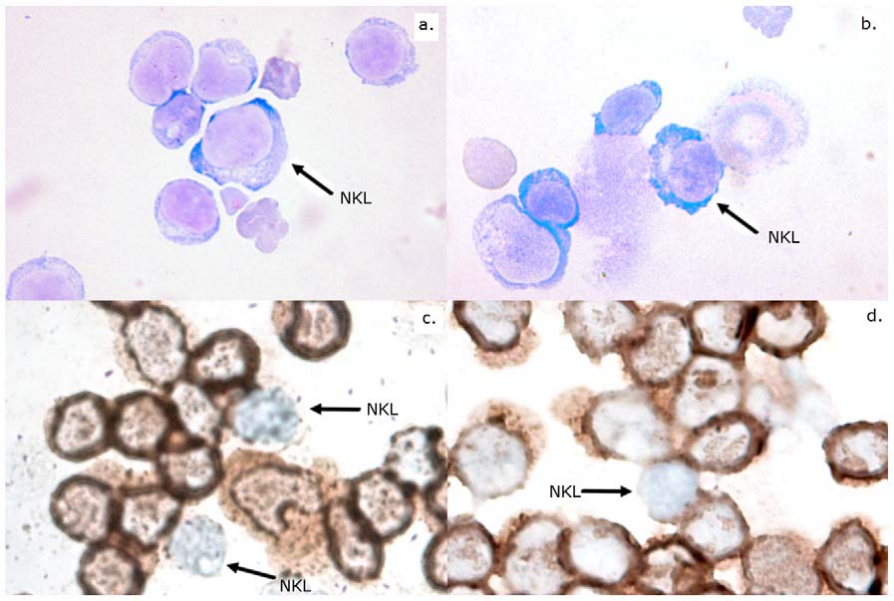
Giemsa staining and immunocytochemistry of Tasmanian devil peripheral blood MNC. Panels a and b show giemsa stained mononuclear cell preparations, in which NK like (NKL) large granular lymphocytes were present. Panels c and d show immunocytochemistry of mononuclear cell preparations with dual staining for CD3 and MHC II. Unlabelled NKL cells can be differentiated from other lymphocytes. Monocytes and neutrophils can be identified by morphology.

Although we have evidence of NK-like ADCC in Tasmanian devils, no previous cytological studies have reported the presence of NK-like cells. The cells will be large granular lymphocytes that do not express MHC II or CD3. Giemsa staining was performed on MNC preparations and large granular lymphocytes were identified (Fig. 8a and c). Antibodies for CD3 and MHC II were then used to co-label mononuclear cell preparations to determine the presence of NK-like cells. Lymphocytes negative for CD3 and MHC II formed approximately 4 percent of the population (Table 1, Fig. 8b and d). This is histological evidence for the presence of NK cells in Tasmanian devils.

## Discussion

Since the discovery of DFTD in 1996, the disease has spread across more than half of Tasmania [1]. Affected Tasmanian devil populations have been devastated by the disease, with numbers in some areas decreasing by 83 percent [14]. Although it is believed that multiple factors are required to cause the demise of a species [15], epidemiological studies estimate that DFTD alone could drive the extinction of the Tasmanian devil in the wild within 25 years [16].

Our work has previously shown that Tasmanian devils have a functional immune system. Although limited reagents are available to analyse the biology of such a little studied species, previous research has confirmed that Tasmanian devils are capable of competent phagocytic responses, lymphocyte proliferation and antibody development [3], [6]. However, there is no evidence of immune responses against DFTD in infected wild devils [3], nor is there lymphocyte infiltration into DFTD tumours [17]. We investigated if a lymphocyte immune response could be forced against DFTD using multiple immunisations of irradiated cells in the presence of adjuvant. Only one devil out of two injected with irradiated DFTD cells formed a cytotoxic response which was weak. With no evidence of antibody development, this reaction is unlikely to be protective against DFTD. Therefore, either the foreign DFTD cells are not detected by the host due to genetic similarity at the major histocompatibility complex (MHC) [18], or Tasmanian devils cannot mount cytotoxic responses.

The development of cytotoxicity responses in Tasmanian devils has not been directly evaluated. We immunised four devils with xenogenic cancer cells (K562, human erythroleukaemia) to induce a maximum cytotoxic response. Most devils immunised with K562 cells developed cytotoxic responses. The responses required prior exposure to the immunogen, which is consistent with a classical cytotoxic T lymphocyte (CTL) response. However, the K562 target cells used in the immunisations were not allogenic and did not express MHC I molecules, the obligatory ligand for CTL activity. Furthermore, enriched populations of T lymphocytes from K562 immunised Tasmanian devils showed reduced cytotoxic activity compared to total mononuclear cells. These factors suggest that CTL activity is not the main effector mechanism in the antitumour responses against K562 in Tasmanaian devils. The cytotoxicity was also dissimilar to classical natural killer (NK) cell killing, as prior exposure was required and the cytotoxicity was only evident after 18 hours. However, irrespective of the mode of action, the identification of functional cytotoxic responses in Tasmanian devils is a promising finding.

As the devils tested also developed antibodies against K562 cells after the second immunisation, a potential mechanism for the responses is antibody dependent cell-mediated cytotoxicity (ADCC). This cytotoxic pathway has been studied in many species [19], [20], [21], [22], [23]. ADCC can be mediated by a variety of innate immune cells, including monocytes,neutrophils [19], [20] and NK cells [23]. It is involved in immune responses including those against viral diseases [20] and cancer [24], [25]. In addition to increasing fractions of T lymphocytes in cell suspensions, filtration through nylon wool depletes antibody producing plasma cells and B lymphocytes [10]. Removal of such cells from the suspensions, and therefore the potential for ADCC responses in the assays may explain the decreased cytotoxicity.

We therefore further examined the involvement of the ADCC pathway in the cytotoxic responses of Tasmanian devils against K562 cells. Initially, the ability of leukocytes from naive Tasmanian devils to form cytotoxic responses in the presence of antibody was assessed using modified cytotoxicity assays containing serum from K562 immunised Tasmanian devils. ADCC responses from naive Tasmanian devils consistently occurred in the presence of serum from immunised devils, suggesting that ADCC was potentially the mechanism that accounted for the cytotoxic responses against K562 cells. Considering the constant exposure to a variety of microbes through the consumption of carrion, the ability to mount efficient antibody responses, along with strong innate responses would be advantageous to a scavenging animal like the Tasmanian devil. Studies performed on other scavenging predators, such as foxes (*Vulpes vulpes*), have indicated that strong antibody responses can be formed against infections acquired from the consumption of infected tissues from prey [26]. We also propose that antibody may be able to influence other facets of the immune system in carrion-feeding animals such as the Tasmanian devil. This would include specific immune responses involved in antitumour immunity. Tasmanian devils are capable of producing strong humoral responses against foreign antigens [6]. It is possible that immunisations targeting antibody development may be important for the induction of cytotoxic responses against DFTD.

Several cell types can act as effectors of ADCC responses, including NK cells, monocytes and neutrophils. By depleting specific populations in mononuclear cell isolates using plastic adherence [11], we characterised the effectors of the ADCC responses in Tasmanian devils. Effective responses were formed by cell suspensions depleted of monocytes following adherence to plastic, thus indicating that monocytes are not the effector cells. The finding that ADCC occurred within four hours further supports the conclusion that CTL, which usually require 18 hours to form *in vitro* cytotoxicity, did not mediate the responses. The mononuclear cell suspensions occasionally contained some neutrophils. However, ADCC responses of neutrophils are only evident at high effector to target ratios [24]. The ADCC assays we performed used lower ratios and neutrophils comprise only a small portion of mononuclear cells. It is therefore unlikely that neutrophils were the effector cells mediating the ADCC.

Since T cells, monocytes and neutrophils can be excluded, NK cells are likely to be the effector cells. NK cells are characterised by unprimed killing but this activity was not observed. However, in the presence of serum from immunised devils, killing occurred within four hours. This rapid killing suggests the functional presence of NK cells. In the absence of specific antibodiesit was not possible to directly identify thesecells. However, cross-species reactive antibodies against CD3 and MHC II have been used to identify cell types in devil lymphoid tissues [27]. Giemsa staining of lymphocytesidentified cells with alarge granular NK-like morphology. Their presence was further implied by immunocytochemistry as lymphocytes negative for both CD3 and MHC II. The combined functional and phenotypic observations provide evidence that functional NK cells exist in Tasmanian devils.

NK cells can contribute to ADCC antitumour responses, such as those induced by monoclonal antibody based human cancer therapies. Drugs like Herceptin, for targeting breast cancer, and Rituximab, for targeting chronic lymphocytic leukaemia and non-Hodgkins lymphoma, are able toinduce NK cytotoxic responses through binding of FcγRIII receptors [25], [28]. Although the Fc receptors of the Tasmanian devil have not yet been characterised, lymphocyte Fc receptors have been identified at the genome level in another marsupial *Monodelphis domestica* [29]. Characterisation of functional Fc receptors in Tasmanian devils will be an important area of future research.

Although there is evidence for a competent immune system in Tasmanian devils, responses against DFTD cells were absent or limited. The fact that DFTD transmission occurs in the presence of a functional immune system suggests a capacity to evade the host antitumour response. Potential mechanisms have been proposed in previous studies. Firstly, there is strong evidence of limited genetic diversity among Tasmanian devils, both in nuclear satellite markers [30] and at the MHC [18]. A low level of genetic diversity may contribute to a lack of immune recognition when infection with the tumour occurs. Secondly, although MHC I and II genes of Tasmanian devils and their RNA transcripts have been identified [31], functional proteins have not been confirmed [32]. If expression of MHC molecules on the membrane of DFTD cells is limited, or the proteins are malformed, DFTD cells may avoid CTL recognition. In this situation, NK cells would be expected to mediate the antitumour immune response.

It is apparent from our study that Tasmanian devils have NK cells capable of producing functional cytotoxic responses in the presence of antibody. It is unknown why NK cells do not directly recognise DFTD cells. However, we have indicated a potential requirement for the presence of specific antibody to mediate their cytotoxicity. This may explain the absence of NK cell activity against DFTD without antibody development. Future studies will aim to induce antibody responses and ADCC against DFTD.

In conclusion, we provide evidence that Tasmanian devils can form functional cytotoxic responses. Although the responses were against xenogenic cells, the involvement of NK cells through the mechanism of ADCC offers a potential pathway to induce a response against DFTD. These are promising findings, with positive implications for DFTD vaccine research.
